# *Coynema* gen. n., a new genus of nematode (Thelastomatoidea, Hystrignathidae) parasites of Passalidae (Coleoptera) from Cuba

**DOI:** 10.3897/zookeys.75.809

**Published:** 2011-01-12

**Authors:** Jans Morffe Rodríguez, Nayla García Rodríguez

**Affiliations:** Instituto de Ecología y Sistemática, Carretera de Varona km 31/2, Capdevila, Boyeros, A.P. 8029, C.P. 10800, Ciudad de La Habana, Cuba

**Keywords:** Nematoda, Hystrignathidae, *Coynema*, Passalidae, *Passalus*, new genus

## Abstract

The new genus Coynema **gen. n.** is described as parasite of the two passalid beetles from Cuba: Passalus interstitialis Escholtz, 1829 (type host) and Passalus pertyi Kaup, 1869. Females are characterized by the shape of their cephalic end, cervical cuticle unarmed, a sub-cylindrical procorpus with its base abruptly dilated, fore region of intestine dilated as a sac-like structure, genital system didelphic-amphidelphic and eggs markedly ovoid and smooth-shelled. Males have a digestive system similar to females, tail sharply pointed, bearing a Y-like thickening of the dorsal cuticle. They also present a big, median, mammiform pre-cloacal papillae and a pair of small, sub-dorsal pre-cloacal papillae anterior to the cuticular thickening of the tail.

## Introduction

The family Hystrignathidae Travassos, 1920 (Oxyurida, Thelastomatoidea) includes about 100 species of parasitic nematodes specific of the gut caeca of passalid beetles. At present, 26 genera are recognized, most of them on the basis of females (Adamson and Van Waerebeke 1992). The shortage of male descriptions is mainly due to the difficulty in assigning specimens to their correct species in co-infections which are common for passalid beetles. The morphological homogeneity of males further contributes to this difficulty.

The Hystrignathidae have a mostly Gondwanian distribution with species in North, Central and South America, West Indies, Africa, Madagascar, Australia and New Guinea. The majority of the genera have been recorded in Brazil (21), most of them being endemic (Travassos and Kloss 1957a, 1957b, 1958; Kloss 1962, Cordeira 1981, Cordeira and Artigas 1983). Some of these genera are also recorded from Mexico (1), Venezuela (2), Saint Lucia (3) and Trinidad (3) (Guerrero 1980, Hunt 1981, 1982, Coy and García 1995, García and Coy 1997).

Australia shows a characteristic fauna of hystrignathids, with the endemic and monotypic genera Anuronema Clark, 1978; Phalacronema Clark, 1978 and Sprentia Clark, 1978 (Clark 1978). Also, Lepidonema Cobb, 1898 and Xyo Cobb, 1898 have been recorded (Cobb 1898).

In Cuba, the study of this group is recent (Coy 1990). Currently, members of the genera Artigasia Christie, 1934; Glaber Travassos & Kloss, 1958; Hystrignathus Leidy, 1850; Lepidonema, Longior Travassos & Kloss, 1958 and Salesia Travassos & Kloss, 1958 are recorded (Coy et al. 1993, García and Coy 1994, 1995a, b, García et al. 2009a, b, c, Morffe et al. 2009, Morffe and García 2010a, b).

Coy et al. (1993) described Glaber poeyi Coy, García &Alvarez, 1993 from Passalus interstitialis. Subsequent examination of type material and specimens collected from other localities demonstrate that these belong to a new genus described in this paper.

## Materials and methods

Five specimens of Passalus pertyi Kaup, 1869 were collected by hand from rotting logs between May, 2007 and March, 2008. Two specimens are from La Melba, Nipe-Sagua-Baracoa, Holguín Province, Cuba, one from El Mulo, Sierra del Rosario, Pinar del Río Province, Cuba and two from La Jaula, San José de las Lajas, La Habana Province, Cuba. Specimens from La Melba and El Mulo were immediately killed and fixed in 70% ethanol. Beetles were dissected by practicing longitudinal incisions in the abdominal pleural membranes and the intestines were extracted and excised in water in Petri dishes under a stereo microscope. The parasites were collected and stored in 70% ethanol.

Beetles from La Jaula were kept alive in plastic jars with moistened wood chips as food and a humidity source. They were killed as soon as possible with ethyl ether vapours and dissected as described above. The intestines were dissected in normal saline instead of water. Nematodes removed from guts were killed with hot water (60–70°C) and fixed in 70% ethanol.

In this study the type material of Glaber poeyi deposited in the Colección Helmintológica of the Colecciones Zoológicas (CZACC), Instituto de Ecología y Sistemática, Havana, Cuba was included.

Nematodes were clear-mounted on slides in glycerine and coverslips were sealed around the edges with nail polish. Measurements were made after Morffe et al. (2009) and are given in millimetres. Variables are showed as range followed by median plus standard deviation and the number of measurements in parentheses. De Man’s ratios a, b, c and V% were calculated.

Micrographs were taken with an AxioCam digital camera attached to a Carl Zeiss AxiosKop 2 Plus compound microscope. Line drawings were made with the softwares CorelDRAW X3 and Adobe Photoshop CS2 using micrographs as templates. Scales of all the plates are given in millimetres.

The materials examined are deposited in the CZACC and the Coleçao Helmintologica do Instituto Oswaldo Cruz (CHIOC), Rio de Janeiro, Brazil.

## Systematics

Family Hystrignathidae Travassos, 1920

### Genus Coynema gen. n.

urn:lsid:zoobank.org:act:3883651B-C4DC-4876-A7D2-1DBAA849E54C

#### Generic diagnosis.

##### Female.

Body robust, markedly fusiform. Cervical cuticle unarmed. Lateral alae present, from the esophageal region to a distance before the level of the vulva. Head bearing eight small, paired papillae. First cephalic annule slightly expanded, set-off from head by a deep single groove, its fore half with concave margins when totally relaxed. Esophagus consisting of a muscular sub-cylindrical procorpus, its base abruptly expanded in its joint with the short isthmus. Intestine simple, its fore region very expanded, forming a sac-like structure. The end of this region of the intestine is abruptly set-off and next to it the gut continues as a simple, rectilinear tube. Nerve ring encircling procorpus at its posterior half. Excretory pore post-bulbar. Reproductive system didelphic-amphidelphic. Eggs large, ovoid, smooth-shelled. Tail long, subulate and filiform.

##### Male.

Body shorter and more slender than female. Cervical cuticle without spines. Head similar to female. First cephalic annule inconspicuous. Digestive system similar to female. Nerve ring encircling procorpus at its posterior half. Excretory pore post-bulbar. One testis present. Spicules absent. Tail conical, very short, sharply pointed, ventrally curved, its dorsal cuticle thickened forming a **Y**-like structure. A single, median, mammiform, big pre-cloacal papilla present. A pair of small, sub-dorsal, pre-cloacal papillae situated before the dorsal thickening.

##### Type species.

Coynema poeyi (Coy, García & Alvarez, 1993) Morffe & García, comb. n. (monotypic genus).

#### Distribution.

Cuba.

#### Etymology.

The generic name is a combination of Coy, after Alberto Coy Otero, eminent Cuban parasitologist and the suffix –nema. The name is in neuter gender.

### Coynema poeyi

(Coy, García & Alvarez, 1993) Morffe & García comb. n.

**Figure 1.**
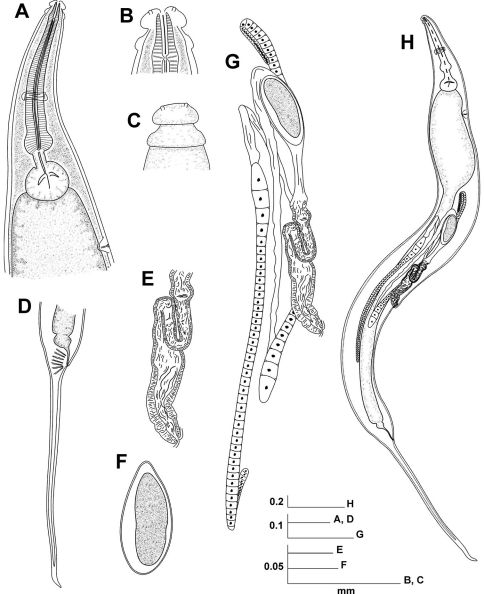
Coynema poeyi (Coy, García & Alvarez, 1993) Morffe & García comb. n. Female. **A** Esophageal region, lateral view **B** Cephalic end, internal view **C** Cephalic end, external view **D** Tail, lateral view **E** Vulva, lateral view **F** Egg **G** Reproductive system **H** Entire nematode, lateral view.

**Figure 2.**
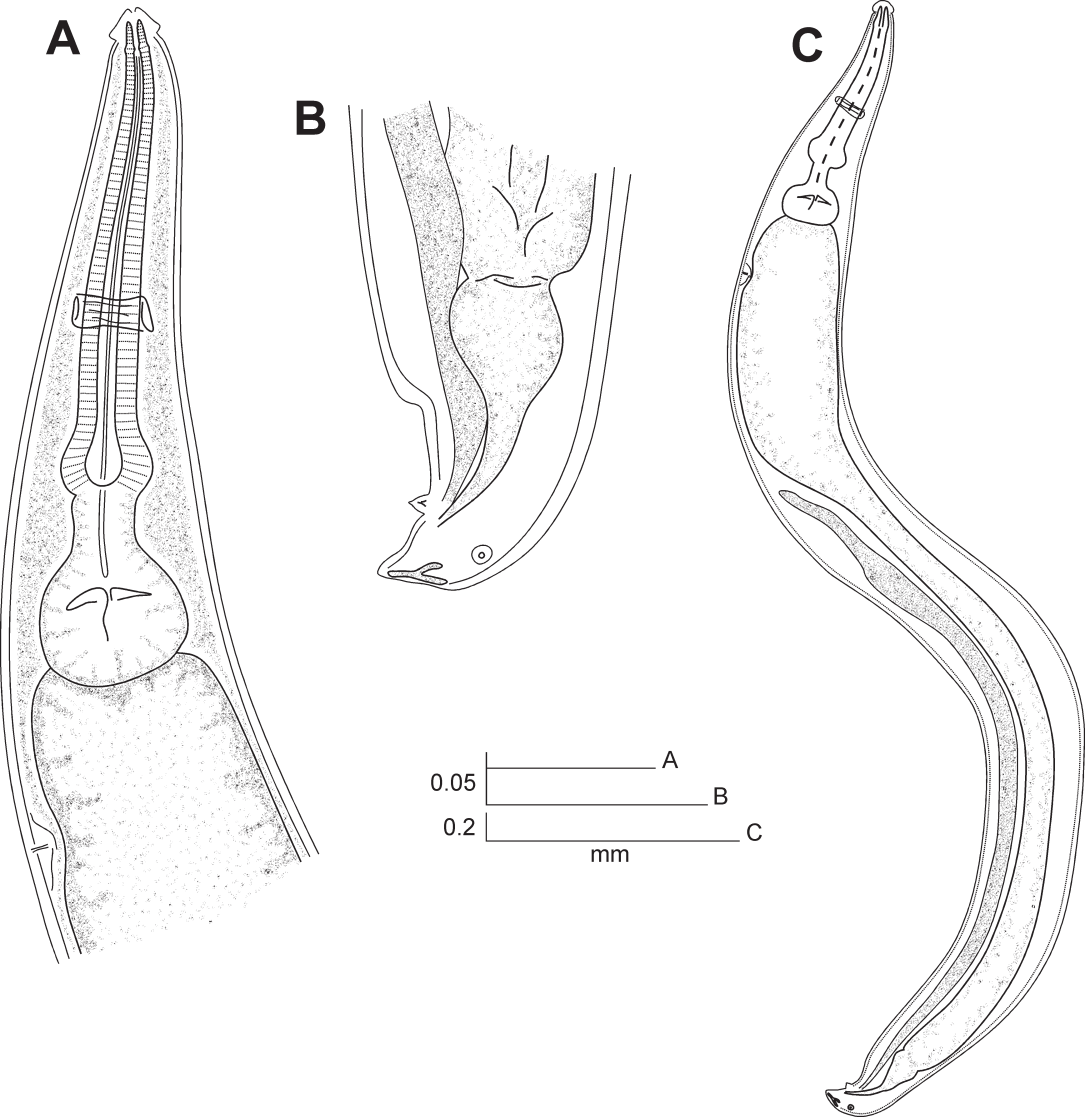
Coynema poeyi (Coy, García & Alvarez, 1993) Morffe & García comb. n. Male. **A** Esophageal region, lateral view **B** Tail end, lateral view **C** Entire nematode, lateral view.

**Figure 3.**
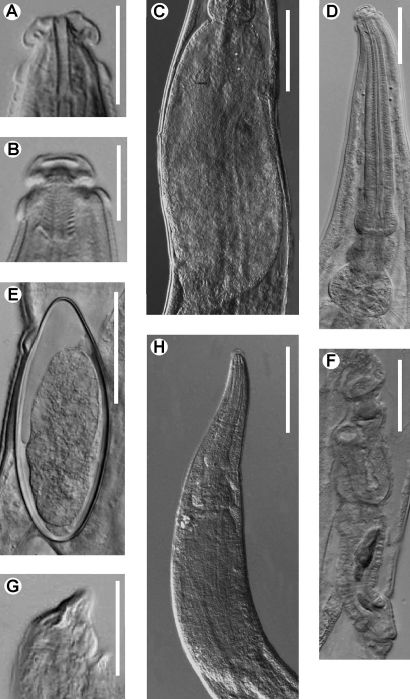
Coynema poeyi (Coy, García & Alvarez, 1993) Morffe & García, comb. n. Female. **A** Cephalic end, relaxed first cephalic annule (specimen killed inside the host) **B** Cephalic end, first cephalic annule not relaxed (heat killed specimen) **C** Sac-like structure of the intestine **D** Esophagus **E** Egg **F** Vulva, lateral view. Male **G** Tail end showing the dorsal cuticular Y-like thickening **H** Esophageal region. Scale bars: **A, B, G** 0.025 mm. **D, E, F** 0.05 mm. **C, H** 0.1 mm.

Figure 1 A–H Figure 2 A–C Figure 3 A–H

Glaber poeyi  Coy, García & Alvarez, 1993: 57–59, fig. 3 A–E

#### Type material.

♂ holotype of Glaber poeyi, Cuba, Pinar del Río Province, Sierra del Rosario, El Salón; in Passalus interstitialis; IV.1990; A. Coy & N. García coll.; CZACC 11.4168. ♀ allotype of Glaber poeyi, same data as holotype, CZACC 11.4169.

#### Other material examined.

5 ♀♀, Cuba, Pinar del Río Province, Sierra del Rosario, El Mulo; in Passalus pertyi; X.2007; R. Núñez & O. Madruga coll.; CZACC 11.4468–11.4472. 9 ♀♀, Cuba, La Habana Province, San José de las Lajas, La Jaula; in Passalus pertyi; 15.III.2008; E. Fonseca, J. Morffe, G. León & F. Alvarez coll.; CZACC 11.4611–11.4619. 2 ♀♀, same data as anterior, deposited in the CHIOC. 6 ♀♀, Cuba, Holguín Province, Nipe-Sagua-Baracoa, La Melba; in Passalus pertyi; V.2007; R. Barba & D. Ortiz coll.; CZACC 11.4460–11.4465. 4 ♂♂, same data as anterior; CZACC 11.4473–11.4476.

#### Type host.

Passalus interstitialis Escholtz, 1829 (Coleoptera, Passalidae).

#### Other host.

Passalus pertyi Kaup, 1869 (Coleoptera, Passalidae).

#### Site.

Gut caeca.

#### Type locality.

El Salón, Sierra del Rosario, Pinar del Río Province, Cuba.

#### Other records.

El Mulo, Sierra del Rosario, Pinar del Río Province, Cuba; La Jaula, San José de las Lajas, La Habana Province, Cuba; La Melba, Nipe-Sagua-Baracoa, Holguín Province, Cuba.

#### Measurements.

Holotype (male) a = 14.40, b = 5.74, c = 83.08, total length = 1.080, maximum body width = 0.075, stoma length = 0.018, procorpus length = 0.130, isthmus length = 0.023, diameter of basal bulb = 0.038, total length of esophagus = 0.188, nerve ring to anterior end = 0.098, excretory pore to anterior end = 0.260, cloaca to posterior end = 0.013.

Allotype (female) a = 16.69, b = 9.15, c = 4.41, V% = 52.63, total length = 2.470, maximum body width = 0.148, first cephalic annule (length×width) = 0.013×0.035, stoma length = 0.025, procorpus length = 0.200, isthmus length = 0.025, diameter of basal bulb = 0.065, total length of esophagus = 0.270, nerve ring to anterior end = 0.130, excretory pore to anterior end = 0.370, vulva to posterior end = 1.170, anus to posterior end = 0.560, eggs = 0.113–0.118×0.048–0.050 (0.116 ± 0.004×0.049 ± 0.001 n = 2).

#### Population from El Mulo, Pinar del Río Province.

Females (n = 5) a = 17.00–19.72 (18.25 ± 1.32 n = 4), b = 7.73–8.65 (8.18 ± 0.38 n = 4), c = 3.65–3.99 (3.82 ± 0.17 n = 4), V% = 46.99–52.94 (49.50 ± 2.84 n = 4), total length = 1.700–2.075 (1.863 ± 0.164 n = 4), maximum body width = 0.090–0.120 (0.102 ± 0.011 n = 5), first cephalic annule (length×width) = 0.008–0.013×0.023–0.030 (0.010 ± 0.002×0.026 ± 0.004), stoma length = 0.018–0.020 (0.019 ± 0.001 n = 5), procorpus length = 0.160–0.175 (0.166 ± 0.006 n = 5), isthmus length = 0.023–0.025 (0.023 ± 0.001 n = 5), diameter of basal bulb = 0.040–0.045 (0.043 ± 0.002 n = 5), total length of esophagus = 0.220–0.240 (0.226 ± 0.009 n = 5), nerve ring to anterior end = 0.108–0.128 (0.119 ± 0.009 n = 5), excretory pore to anterior end = 0.340 (0.340 n = 1), vulva to posterior end = 0.800–1.100 (0.944 ± 0.133 n = 4), anus to posterior end = 0.430–0.520 (0.484 ± 0.038 n = 5), eggs = 0.105–0.113×0.035–0.048 (0.110 ± 0.003×0.040 ± 0.003 n = 11).

#### Population from La Jaula, La Habana Province.

Females (n = 11) a = 13.13–20.00 (16.26 ± 1.95 n = 11), b = 7.65–9.33 (8.35 ± 0.53 n = 11), c = 3.93–4.49 (4.22 ± 0.19 n = 10), V% = 47.72–51.92 (50.44 ± 1.07 n = 11), total length = 1.740–2.425 (2.010 ± 0.211 n = 11), maximum body width = 0.108–0.150 (0.125 ± 0.014 n = 11), first cephalic annule (length×width) = 0.005–0.008×0.028–0.033 (0.007 ± 0.001×0.029 ± 0.002 n = 7), stoma length = 0.020 (0.020 n = 11), procorpus length = 0.163–0.185 (0.174 ± 0.008 n = 11), isthmus length = 0.020–0.028 (0.023 ± 0.003 n = 11), diameter of basal bulb = 0.043–0.058 (0.049 ± 0.005 n = 11), total length of esophagus = 0.228–0.260 (0.240 ± 0.010 n = 11), nerve ring to anterior end = 0.120–0.140 (0.130 ± 0.006 n = 11), excretory pore to anterior end = 0.280–0.360 (0.320 ± 0.029 n = 11), vulva to posterior end = 0.860–1.200 (0.996 ± 0.107 n = 11), anus to posterior end = 0.400–0.560 (0.478 ± 0.061 n = 10), eggs = 0.108–0.125×0.035–0.048 (0.116 ± 0.004×0.042 ± 0.004 n = 20).

#### Population from La Melba, Holguín Province.

Females (n = 6) a = 13.27–16.00 (14.80 ± 0.95 n = 6), b = 6.77–8.38 (7.53 ± 0.57 n = 6), c = 3.35–3.84 (3.60 ± 0.20 n = 6), V% = 43.18–48.24 (44.92 ± 2.27 n = 4), total length = 1.460–1.800 (1.653 ± 0.126 n = 6), maximum body width = 0.110–0.120 (0.112 ± 0.004 n = 6), first cephalic annule (length×width) = 0.010×0.025–0.028 (0.010×0.027 ± 0.001 n = 5), stoma length = 0.018–0.020 (0.018 ± 0.001 n = 6), procorpus length = 0.153–0.173 (0.160 ± 0.008 n = 6), isthmus length = 0.018–0.200 (0.019 ± 0.001 n = 6), diameter of basal bulb = 0.043–0.065 (0.048 ± 0.009 n = 6), total length of esophagus = 0.200–0.240 (0.220 ± 0.014 n = 6), nerve ring to anterior end = 0.113–0.125 (0.119 ± 0.006 n = 6), excretory pore to anterior end = 0.270–0.360 (0.307 ± 0.047 n = 3), vulva to posterior end = 0.820–1.000 (0.894 ± 0.076 n = 4), anus to posterior end = 0.380–0.530 (0.462 ± 0.052 n = 6), eggs = 0.105–0.125×0.035–0.048 (0.113 ± 0.006×0.043 ± 0.004 n = 11).

Males (n = 4) a = 10.84–13.18 (11.87 ± 1.05 n = 4), b = 5.62–6.03 (5.88 ± 0.18 n = 4), c = 55.00–74.67 (66.92 ± 8.39 n = 4), total length = 1.030–1.120 (1.073 ± 0.044 n = 4), maximum body width = 0.085–0.095 (0.091 ± 0.004 n = 4), stoma length = 0.015–0.020 (0.017 ± 0.002 n = 4), procorpus length = 0.125–0.300 (0.128 ± 0.002 n = 4), isthmus length = 0.015–0.023 (0.018 ± 0.004 n = 4), diameter of basal bulb = 0.040–0.048 (0.044 ± 0.003 n = 4), total length of esophagus = 0.175–0.188 (0.183 ± 0.005 n = 4), nerve ring to anterior end = 0.093–0.100 (0.096 ± 0.003 n = 4), excretory pore to anterior end = 0.240–0.250 (0.248 ± 0.005 n = 4), cloaca to posterior end = 0.015–0.020 (0.016 ± 0.003 n = 4).

#### Description.

##### Female.

Body comparatively robust, markedly fusiform, maximum width at level of the anterior part of intestine. Cervical cuticle unarmed, finely annulated (annule more conspicuous toward post-esophageal region). Sub-cuticular longitudinal striae present. Lateral alae from the end of procorpus or the beginning of its basal dilation to about half of a body width before the level of vulva. Head well developed, set-off from body by a single, deep groove and bearing eight small paired papillae. First cephalic annule slightly expanded, when totally stretched (in dead nematodes inside the hosts) consisting of an anterior half with concave margins (its diameter initially inferior to that of head and increasing gradually in the posterior direction) and a posterior half wider and with convex margins. In heat-relaxed specimens the cephalic annule appears to be less stretched and only the posterior part is visible. Stoma short, about two first cephalic annule lengths long, surrounded by an esophageal collar. Esophagus consists of a muscular, sub-cylindrical procorpus, base abruptly dilated in its joint with the short isthmus. Basal bulb pyriform, valve plate well developed. Intestine simple, sub-rectilinear, anterior portion notably dilated, forming a sac-like structure slightly longer than esophagus. In this part the external surface of intestine almost touching the body wall. The continuation of intestine is about one third of the diameter of the sac-like structure. Rectum short, anus not prominent. Nerve ring encircling procorpus at about 60% of its length. Excretory pore situated at about half of a body width posterior to basal bulb. Genital tract didelphic-amphidelphic, both ovaries thin, reflexed. Anterior ovary commencing just behind the saccular structure and posterior ovary arising slightly more than a body width anterior to the level of anus. Vulva a median transverse slit near midbody or slightly displaced forward, lips more or less prominent. Vagina muscular, thin-walled, forwardly directed. Conduct next to the vagina forming a loop. Eggs comparatively large, markedly ovoid in shape, smooth-shelled. Tail comparatively long, filiform and subulate.

##### Male.

Body shorter and slender than female. Cervical cuticle unarmed. Sub-cuticular longitudinal striae present. Cephalic end similar to female, except by the cephalic annule inconspicuous. Digestive system similar to female. Sac-like region of the intestine slightly larger than the esophagus. Rectum short and cloaca inconspicuous, not prominent. Nerve ring encircling procorpus in posterior half, at about 60% of its length. Excretory pore situated at about less than a body-width posterior to the basal bulb. Testis single, commencing just posterior to the sac-like structure of intestine. Tail conical, sharply pointed, very short and ventrally curved. Dorsal cuticle near the tail tip bearing a Y-like thickening, its inferior part posteriorly directed. A single, median, large, mammiform pre-cloacal papilla present. A pair of small, sub-dorsal, pre-cloacal papillae situated before the dorsal cuticular thickening. Spicules absent.

#### Discussion.

Coynema gen. n. can be placed in Hystrignathidae by having males with the single median pre-cloacal papilla characteristic of the family, oesophagus with its anterior portion supported by cuticularized rods and elongated eggs (Adamson and Van Waerebeke 1992). The new genus has affinities with the Brazilian genus Glaber by having a similar arrangement and form of the first cephalic annule and the characteristic basal dilation of the procorpus. A similar procorpus is present in Vulcanonema Travassos & Kloss, 1958, that however differs in its first cephalic annule separated from head by a conical region. These last two genera can be differentiated from Coynema gen. n. in having a monodelphic-prodelphic reproductive system. The marked fusiform shape of Coynema gen. n. only appears in the monotypic Malagasian genus Passalidophila Van Waerebeke, 1973, which is also monodelphic.

There are eight hystrignathid genera that are digonant and lack spines in the cervical cuticle: Anomalostoma Cordeira, 1981; Anuronema, Klossnema Cordeira & Artigas, 1983; Papillabrum Cordeira, 1981; Phalacronema, Sprentia, Triumphalisnema Kloss, 1962 and Ventelia Kloss, 1962. Coynema gen. n. differs from all of them in the form of cephalic end, the esophagus and the sac-like structure of the intestine. At present, the latter feature appears to be unique in the family.

The male of Coynema gen. n. presents the head and digestive system very similar to female. This is unusual in the few genera of Hystrignathidae having males described, which have a cylindrical procorpus. Due to this, the shape of the digestive system differentiates the males of Coynema gen. n. from the other males of Hystrignathidae.

#### Comments.

There are small metric differences in the average values among females from some of the populations studied. In spite of this measurements tend to overlap, and there were no evident morphological differences observed. Males from El Salón and La Melba did not show marked morphometric variation. In addition, no male specimens were found in the populations from El Mulo and La Jaula, when looking for more features that would support the existence of other species of Coynema gen. n. These are the reasons why all populations are considered conspecific until larger series of specimens are at hand for further study.

## Supplementary Material

XML Treatment for Coynema

XML Treatment for Coynema poeyi
